# Nuclear and mitochondrial genetic structure in the Eurasian beaver (*Castor fiber*) – implications for future reintroductions

**DOI:** 10.1111/eva.12162

**Published:** 2014-06-17

**Authors:** Helen Senn, Rob Ogden, Christiane Frosch, Alena Syrůčková, Roisin Campbell-Palmer, Pavel Munclinger, Walter Durka, Robert H S Kraus, Alexander P Saveljev, Carsten Nowak, Annegret Stubbe, Michael Stubbe, Johan Michaux, Vladimir Lavrov, Ravchig Samiya, Alius Ulevicius, Frank Rosell

**Affiliations:** 1 WildGenes Laboratory, Royal Zoological Society of ScotlandEdinburgh, UK; 2 Conservation Genetics Group, Senckenberg Research Institute and Natural History Museum FrankfurtGelnhausen, Germany; 3 Department of Zoology, Faculty of Science, Charles University in PraguePrague, Czech Republic; 4 Department of Community Ecology, Helmholtz Centre for Environmental Research – UFZHalle, Germany; 5 Russian Research Institute of Game Management and Fur Farming, Russian Academy of SciencesKirov, Russia; 6 Martin-Luther-Universität Halle-Wittenberg Institut für Biologie Bereich Zoologie/Molekulare Ökologie Hoher Weg 4Halle/Saale, Germany; 7 Martin-Luther-Universität Halle-Wittenberg Institut für Biologie Domplatz 4Halle/Saale, Germany; 8 Conservation Genetics Unit, Institute of Botany (Bat. 22), University of Liège (Sart Tilman)Liège, Belgium; 9 Voronezh State Biosphere ReserveVoronezh, Russia; 10 Department of Zoology, School of Biology and Biotechnology, National University of MongoliaUlaanbaatar, Mongolia; 11 Faculty of Natural Sciences, Vilnius UniversityVilnius, Lithuania; 12 Telemark University College, Department of Environmental SciencesTelemark, Norway

**Keywords:** applied conservation genetics, ascertainment bias, evolutionarily significant unit, inbreeding depression, outbreeding depression, population augmentation, conservation genomics

## Abstract

Many reintroduction projects for conservation fail, and there are a large number of factors that may contribute to failure. Genetic analysis can be used to help stack the odds of a reintroduction in favour of success, by conducting assessment of source populations to evaluate the possibility of inbreeding and outbreeding depression and by conducting postrelease monitoring. In this study, we use a panel of 306 SNP (single nucleotide polymorphism) markers and 487–489 base pairs of mitochondrial DNA control region sequence data to examine 321 individuals from possible source populations of the Eurasian beaver for a reintroduction to Scotland. We use this information to reassess the phylogenetic history of the Eurasian beavers, to examine the genetic legacy of past reintroductions on the Eurasian landmass and to assess the future power of the genetic markers to conduct ongoing monitoring via parentage analysis and individual identification. We demonstrate the capacity of medium density genetic data (hundreds of SNPs) to provide information suitable for applied conservation and discuss the difficulty of balancing the need for high genetic diversity against phylogenetic best fit when choosing source population(s) for reintroduction.

## Introduction

At least a third of reintroduction projects for conservation purposes fail (Fischer and Lindenmayer 2000; Germano and Bishop 2009; Godefroid et al. 2011). There are a large number of reasons for failure, but these can often only be guessed at because of poor monitoring or follow-up (Fischer and Lindenmayer 2000). Factors that could be assessed and monitored through genetic tools such as inbreeding, outbreeding/hybridization and loss of genetic diversity leading to loss of adaptive potential are commonly cited as putative reasons for reintroduction failure (Frankham 1995; Marshall and Spalton 2000; Kephart 2004; Tallmon et al. 2004; Vilas et al. 2006; Weeks et al. 2011). For this reason, the ability to properly assess and monitor the genetic components of a reintroduction project is vital (IUCN 1998, 2013; Seddon et al. 2007). Genetic analysis has been used in relation to reintroductions in a number of ways, from selection of founders and ongoing monitoring to surveying of the genetic impact of reintroduction, many years after unmonitored release (e.g. Marshall and Spalton 2000; Latch and Rhodes 2006; Grueber and Jamieson 2007; Wisely et al. 2007; Ewing et al. 2008; De Barba et al. 2010; Koelewijn et al. 2010; El Alqamy et al. 2011; Kim et al. 2011; Ozer et al. 2011; Cullingham and Moehrenschlager 2013; Shephard et al. 2013; Tollington et al. 2013). Ideally genetic information should be taken into consideration both in the planning and monitoring phases; however, to date, many reintroduction projects have been hampered by a lack of appropriate genetic resources (markers) and baseline genetic data for the target species (Allendorf et al. 2010). Species of conservation concern were not traditionally the subject of detailed genomic studies and have, in the past, relied on cross-fostering of genetic resources from closely related species of interest to medicine and agriculture. This is now changing due to the ever-increasing number of whole-genome sequencing studies of nonmodel species (Haussler et al. 2009) and due to the advent of reduced representation/next-generation/genotype-by-sequencing technologies (Narum et al. 2013). Although, in this age of rapidly developing genetic technology, slow information transfers from academic genetics to truly applied conservation can also hinder progress. In conservation, there is often a tension between waiting for research to generate answers and acting before it is too late. Here, in a study conducted in support of the reintroduction of the Eurasian beaver, *Castor fiber*, to Scotland, we demonstrate the capacity of medium -density SNP (hundreds of single nucleotide polymorphisms) genotyping derived from RAD sequencing data (Senn et al. 2013), to deliver genetic information appropriate for planning and monitoring a reintroduction.

### Castor fiber

**Figure 1:**
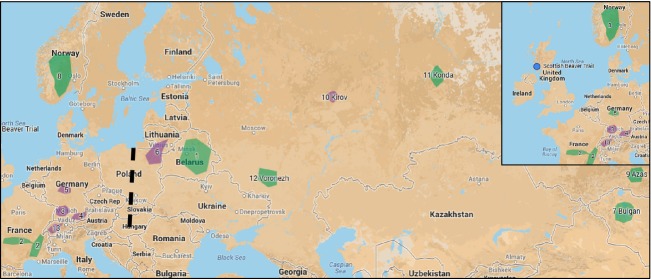
A map of the sample locations. Fur trade (FT) refugial populations sampled are coloured in green. Other populations are coloured in purple. The location of the Scottish Beaver trial is given in the top right inset. The numbered populations are as in Table 2: 1. Belarus (*belorussicus* FT refugia) 2. France (*galliae* FT refugia), 3. Germany: Baden-Württemberg (reintroduced), 4. Germany: Bavaria (reintroduced), 5. Germany: Hesse (reintroduced *albicus*), 6. Lithuania and Poland (reintroduced), 7. Mongolia: Bulgan (*birulai* FT refugia) 8. Norway (*fiber* FT refugia), 9. Russia: Azas (*tuvinicus* FT refugia). 10. Russia: Kirov (reintroduced) 11. Russia: Konda (*pohlei* FT refugia) 12. Russia: Voronezh (*orientoeuropaeus* FT refugia). The dashed line gives the approximate location of the putative boundary between eastern and western ESU.

The Eurasian beaver can be seen as a European and Asian conservation success. Driven to virtual extinction by the fur trade in the 19th century, the species now inhabits large tracts of its former range as a result of the cessation of hunting, followed by a number of reintroductions and natural expansions from relict populations. Detailed descriptions of population range and history have been published elsewhere (Macdonald et al. 1995; Nolet and Rosell 1998; Halley and Rosell 2002, 2003; Durka et al. 2005; Dewas et al. 2012; Halley et al. 2012). By the start of the 1900's only a few relict populations survived having passed through bottlenecks of between 30 and 300 individuals (Table 1). These relict populations have traditionally been given subspecific status based on cranial morphometrics (Freye 1960; Lavrov 1979; Heidecke 1986; Frahnert 2000; Table 1, Fig. 1). DNA evidence from mitochondrial DNA (mtDNA) and MHC DRB gene sequences reveals that these populations are characterized by low genetic diversity and pronounced genetic structuring (Ellegren et al. 1993; Babik et al. 2005; Durka et al. 2005). Based on analysis of the mtDNA control region, a lineage division exists within *C. fiber* for two apparently reciprocally monophyletic clades which correspond to Rhône (France ssp. *galliae*), Telemark (Norway, ssp. *fiber*) and Elbe (Germany, ssp. *albicus*) fur trade (henceforth FT) refugia in the west, and the Voronezh (Russia, ssp. *orientoeuropaeus*), Konda (Russia, ssp. *pohlei*), Azas (Russia, ssp. *tuvinicus*) and Bulgan (Mongolia, ssp. *birulai*) FT refugia in Eastern Europe and central Eurasia (Durka et al. 2005; Horn et al. 2014). Most recent common ancestor for the two clades has been estimated to be 210 000 (110 000–340 000) years old (Horn et al. 2011), a timing corresponding to the previous interglacial (i.e. before last glacial maxima). Durka et al. (2005) have proposed these two clades as evolutionary significant units (ESU) and have suggested that reintroductions should not mix western and eastern ESU stocks.

**Table 1:** The main early 20th century fur trade (FT) refugia of Eurasian beaver and traditional subspecific status associated with them. These are the FT refugia from which current Eurasian beaver population are thought to have become re-established. There were still undoubtedly a number of other FT refugial population in existence in the early half of the 20th century in Poland (Dzieciolowski and Gozdziewski 1999), Turkey (Kogan 1933), Kazakhstan and Russian Siberia; however, these population are thought to have become extinct

	Subspecies classification*	Associated FT refugia	Population bottleneck size	Reference for population size	Durka et al. (2005) ESU classification based on mtDNA cytB
1	*Castor fiber galliae*	Lower Rhône, France	30	Richard (1985)	Western ESU
2	*C. f. fiber*	Telemark, Norway	60–120	Collett (1897)	
3	*C. f. albicus*	Elbe, Germany	200	Heidecke and Hörig (1986)	
4	*C. f. belorussicus*†	Dnepr and Neman river basins Lithuania/Belarus/Ukraine/Russia‡	<300	Dehnel (1948), Serzhanin (1949)	Unknown
5	*C. f. orientoeuropaeus*§	Voronezh, Russia	70	Lavrov and Lavrov (1986)	Eastern ESU
6	*C. f. pohlei*¶	Konda, Russia	300	Lavrov and Lavrov (1986)	
7	*C. f. tuvinicus*	Azas, Russia	30–40	Lavrov and Lavrov (1986)	
8	*C. f. birulai*¶	Bulgan, Mongolia/China	<100–150	Lavrov and Hao-Tsuan (1961)	

* Following preferred classification in Durka et al. (2005), see references therein.

† Also referred to as *C. f. belarusicus*.

‡ The so-called *belorussicus* FT refugium in fact consisted of FT refugia in two separate river systems (Neman and Dnepr) that may have been completely unrelated (Dehnel 1948; Serzhanin 1949).

§ Also referred to as *C*. *f. osteuropaeus*.

¶ It has been argued that *pohlei* and *birulai* should never have been classed as separate (Saveljev et al. 2011). Although situated >2000 km apart, the populations were both part of the Irtysh river system and most likely formed a single continuous population 90–100 years ago.

### Conservation status

*Castor fiber* is currently listed as Least Concern by the International Union for the Conservation of Nature (IUCN) and it is listed under the Bern Convention (Appendix III) and the EU Habitats and Species Directive (Annex V for the Swedish and Finnish populations, Annex II and IV for all others). Population estimates for Eurasia are around 1 million individuals (Halley et al. 2012), although locally they can exist in low numbers or be absent from suitable habitat. As highlighted above, genetic studies conducted to date (Babik et al. 2005; Durka et al. 2005) imply that high population numbers are underpinned by low levels of genetic diversity; unsurprising given what we know of the population history. This lack of diversity is relevant to the continued conservation of the Eurasian beaver as it may cause reduced potential for the populations to adapt to future conditions, for example disease or environmental change. The beaver is a keystone species in the Eurasian riparian ecosystem, because through dam building it alters stream hydrology and morphology and so has considerable influence on surrounding animal and plant communities (Nummi and Pöysä 1997; Nummi and Hahtola 2008; Nummi et al. 2011).

### Reintroductions

There have been numerous beaver reintroductions and augmentations across Eurasia, the histories of which are complicated, and documentation is often absent (Macdonald et al. 1995; Nolet and Rosell 1998; Dzieciolowski and Gozdziewski 1999; Halley and Rosell 2002; Halley et al. 2012). A number of reintroductions have involved multiple population sources, sometimes mixing animals from the postulated eastern and western ESUs (Durka et al. 2005). So far reintroductions have been carried out with little reference to genetic data. There has also, until recently, been limited genetic follow-up (Horn et al. 2010; Frosch et al. 2014) to assess the extent to which different founders have contributed to the reintroduction.

One of the most recent examples of a planned Eurasian beaver reintroduction is the Scottish Beaver Trial, the first trial reintroduction of the species to Britain (Table 2). The project commenced in 2009 and the initial stage, a trial (with a very limited number of monitored individuals) lasting until 2015, is designed to assess the feasibility of a full reintroduction. Britain is isolated from the Eurasian landmass, so natural recolonization and joining of different reintroduced populations, in the way that has happened in a number places in Europe (see Table 2), is not possible. Once reintroduction has taken place, no natural gene flow can occur from other populations. For this reason in particular, the careful consideration of the founder population and the ongoing monitoring of the reintroduced stock were considered to be important tasks for the reintroduction (Rosell et al. 2012).

**Table 2:** Sample locations and purported origins of the animals from those locations. The locations are mapped on Fig. 1

	Sample location	Purported genetic origin of population*	*n* (chip)	*n* (>95% of SNPs amplified)
1	Belarus (Dnepr and Neman river basins)	*belorussicus* FT refugia	30	24
2	France	*galliae* FT refugia	18	11
3	Germany: Baden-Württemberg	Reintroduction from *galliae* FT refugia	15	15
4	Germany: Bavaria	Mixed reintroduction [*fiber*, *belorussicus*, *galliae*, and probably also *orientoeuropaeus*]	49	48
5	Germany: Hesse	Reintroduction from the *albicus* FT refugia on the Elbe with likely incursions from neighbouring Bavarian origin populations	16	16
6	Lithuania and Poland	Mixed reintroduction of *orientoeuropaeus* and *belorussicus*	42	40
7	Mongolia: Bulgan	*birulai* FT refugia	12	5
8	Norway	*fiber* FT refugia	60	48
9	Russia: Azas	*tuvinicus* FT refugia	15	11
10	Russia: Kirov	*orientoeuropaeus* and *belorussicus* (Dnerp)	11	10
11	Russia: Konda	*pohlei* FT refugia	10	10
12	Russia: Voronezh	*orientoeuropaeus* FT refugia	17	16
13	Switzerland	mixed [*fiber*, *galliae*, *belorussicus* and *orientoeuropaeus*]	26	25
*–*	*Castor canadensis*	Samples from USA and Germany (zoo escapees)	5	(0)†

* From Halley and Rosell (2002), Macdonald et al. (1995), Nolet and Rosell (1998) and unpublished information gathered from authors.

† See text for details of loci that cross-amplified to this species.

At the commencement of the trial, the genetic resources for beavers were insufficient. A number of microsatellite markers for the North American beaver (*Castor canadensis*) were available but few cross-amplify successfully to the Eurasian beaver (Crawford et al. 2008; Pelz-Serrano et al. 2009). A later study (Frosch et al. 2010) isolated 15 markers in Eurasian beaver, which although polymorphic tend to show fixed differences between populations and their utility for parentage and intrapopulation analysis is limited to some populations. To address this resource gap, partial genome sequencing of eight beavers from Germany and Norway was conducted using paired-end restriction-site-associated DNA (PE-RAD) sequencing (Baird et al. 2008; Etter et al. 2011), resulting in the discovery of 6637 SNPs (Senn et al. 2013). This study now reports on a pan-Eurasian survey of *C. fiber* at 306 of these SNP loci discovered by Senn et al. (2013).

The aims of this study were:

1. To re-evaluate the Eurasian beaver phylogeny and ESU concept by inclusion of previously undersampled FT refugial areas and nuclear data.
2. To gain the first comprehensive comparison of nuclear genetic diversity across Eurasia. What are the comparative levels of genetic diversity in FT refugial populations and what is the degree of admixture among European beaver populations? What is their relative suitability as source populations for future reintroductions?
3. To examine the power of smaller panels of SNP markers for conducting *in situ* monitoring for management of reintroduced beavers.

The first two points will serve as a baseline for the choice of individuals for future reintroductions, while the third point may help to establish a rapid genetic screening system for applied reintroduction genetics of beavers. This study will not only inform any future Scottish reintroduction, but also provide data to inform further reintroductions to the rest of Europe.

## Methods

### Sample collection and DNA extraction

Samples of beaver were collected from throughout Europe and Asia (for a full list see Fig. 1 and Table 2), consisting of 321 samples of *C. fiber* from 13 FT refugial and reintroduced populations across Eurasia and five samples of the Canadian beaver *C. canadensis* (from individuals living in the USA and Europe). The *C. canadensis* samples were included in order to assess cross-amplification and polymorphisms in this closely related species. Samples came from blood stored in EDTA or a variety of tissue types (stomach, liver, muscle) and were extracted using the DNeasy blood and tissue kit (Qiagen; http://www.qiagen.com). DNA was quantified using a Nanodrop 1000 (Thermo Scientific; http://www.thermoscientific.com) and was normalized to between 35 and 50 ng/*μ*l concentration.

### Assay design

Using the SNP data generated by Senn et al. (2013), a 384 SNP Illumina Veracode assay was designed. SNPs were selected for the assay according to the following criteria: 1. they had previously been shown to be polymorphic in multiple individuals and had high coverage [criteria detailed in Senn et al. (2013)]; 2. they had a minimum of 100 bp of flanking region on each side to facilitate future assay design; and 3. they had Illumina assay design scores of >0.9.

The SNP discovery phase (Senn et al. 2013) was conducted on ten individuals from two populations in Norway and a population in Bavaria. A number of criteria were added for markers included in the assay in addition to the criteria of Senn et al. (2013): 25% (96) of the markers were selected because they showed polymorphism in the Bavarian samples (regardless of status in Norway), 25% (96) were selected because they showed polymorphism in Norwegian samples (regardless of status in Bavaria), 10% (39) were selected that showed apparent fixed differences between Bavaria and Norway, and 40% (153) were selected at random from the remaining markers. These criteria were imposed so as to ensure the utility of a subset of the SNP markers in future for individual and parentage identification in both Bavarian and Norwegian populations, but also to have, in the randomly selected SNPs, a subpanel without additional bias (however, see Discussion of the possible effects of ascertainment bias later).

### Genotyping

SNP genotyping was conducted by ARK Genomics (Roslin, UK) on a BeadXpress System (Illumina; http://www.illumina.com). DNA samples were placed in a randomized order, and negative controls and positive controls were placed on each plate as standard. Genotype scoring was conducted using Genome Studio (Illumina). After initial clustering of the SNP data, individuals that showed a low call rate (<95% of the SNPs) were rejected and the data were clustered excluding these individuals. All clusters called were manually inspected and adjusted by eye if necessary.

### Analysis methods

Analyses were performed on two data sets: all loci, that is, the entire data set of 306 SNPs which remained once monomorphic and failed loci were excluded; and the reduced set of 104 randomly selected SNPs. This reduced set of SNPs was used for the Structure analysis (see below).

Basic population genetic statistics, such as Hardy–Weinberg, heterozygosity, allelic richness, Fst, and the assessment of the markers’ power for individual ID and parentage testing were conducted in Genalex version 6 (Peakall and Smouse 2006).

Analysis of genetic population structure was conducted using two methods, first via principal component analysis (PCA) in Genalex 6 to gain a general overview of the structure of the data set. Principle component analysis has been shown to have a high power of population assignment (Patterson et al. 2006), and data interpretation is straight forward, although it is not quantitative with respect to grouping.

The second method was Bayesian clustering in STRUCTURE 2.3.4 (Pritchard et al. 2000; Falush et al. 2007) to examine population structure and to assess admixture. The algorithm assigns individuals to one or more of *K* populations which as far as possible conform to Hardy–Weinberg and linkage equilibrium. Significant membership to multiple populations (e.g. >5%, Senn and Pemberton (2009)) is interpreted as admixture: The model was run using a burn-in of 5 × 10^5^ and a run of 10^6^ Markov chain Monte Carlo steps, under the standard model of admixed ancestry (with the parameter alpha inferred from the data, using a uniform prior) and the model of correlated allele frequency (*λ* = 1). Ten independent replicates of *K* = 1–20 were conducted. How to choose which value of *K* is biologically meaningful remains a subject to debate (Evanno et al. 2005; Pritchard 2010). In general, it should be possible to subdivide genotype data sets meaningfully to a number of different ‘population’ levels (equivalent to branches on a tree) and so to talk of the existence of a ‘true *K*’ for a given data set is not particularly helpful. Here, the most biologically meaningful value of *K* was determined to be the lowest value that captured the data structure – as with all statistical model choices the best model is the one that describes the data with the fewest parameters. We quantified this by selecting the lowest value of *K* where the posterior likelihood Ln(PD) was not significantly lower than the *K* + 1'th value (determined by Wilcoxon rank-sum as in Willing et al. (2010)). As the choice of *K* cannot escape subjectivity, we additionally provide results for other values of *K*. Because no data for physical genetic distances between the SNPs are available, we employed the standard STRUCTURE model for unlinked markers (as in Kraus et al. (2013)). Using the smaller panel of 104 ‘random’ SNPs (see above) should violate assumptions of nonlinkage to a lesser degree, although in fact, there was little difference between the two data sets. Here, we present, for the STRUCTURE analysis, only the results from the random 104 SNPs.

**Figure 2:**
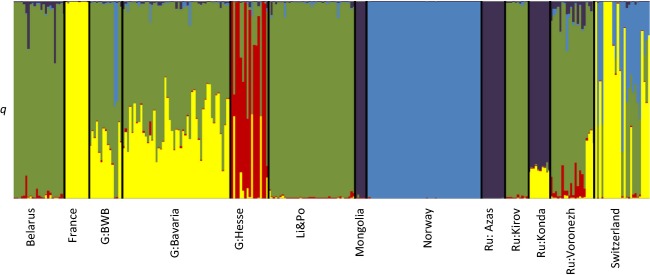
The probability of each individual belonging to each of genetic clusters (STRUCTURE's Q). Analysis based on 104 SNPs. Each vertical line represents a single individual. The animals divide into five clusters blue = Norway, yellow = France, red = Albicus, green = Eastern Europe, purple = Central Eurasia.

To examine the evolutionary relationship between the FT refugial beaver populations, a phylogenetic network of the SNP data was assembled using the method neighbour-net (Bryant and Moulton 2004) in SPLITSTREE4 (Huson and Bryant 2006). We conducted the analysis by compiling an artificial haploid nucleotide sequence of all 306 SNPs with heterozygous SNPs coded as degenerate bases according to IUPAC, and missing data coded as N. Ambiguous (heterozygous) states were handled as average matches. As the intention of this analysis was to examine the evolutionary relationship, the tree was only built for individuals from known FT refugial populations. In the case of the animals from Hesse, only those belonging predominantly to cluster 4 were selected (Fig. 2) under the assumption that these represented the nonintrogressed ‘*albicus* type’.

### MtDNA sequencing and analysis

Sequences of 487–489 bp of mtDNA control region haplotype were available (Durka et al. 2005) or were generated according to Durka et al. (2005), for a subset of 222 of the genotyped animals, with individuals representing all populations. Novel haplotypes were submitted to GenBank under accession numbers KJ670496–9. Sequences were edited and aligned in Geneious version 6.1.5 (Biomatters; http://www.biomatters.com), with final correction done by eye. Sequences were aligned to the GenBank sequence NC_015108.1 (Horn et al. 2011) from *C. canadensis* which was used as an out-group.

Tree building using a basic distance method was conducted via the neighbour-joining method in Geneious version 6.1.5 using the Tamura–Nei genetic distance model.

Bayesian inference of phylogeny was conducted in MrBayes version 3.2.1 (Huelsenbeck and Ronquist 2001) using an HKY85 substitution model with gamma model for rate variation. Analysis was conducted for 1 100 000 MCMC replicates with a burn-in of 100 000 MCMC replicates and subsampling every 200 trees over four replicates.

The alignment was visualized using the median-joining network (Bandelt et al. 1999) produced in Network 4.6.1.1 (Fluxus Technology Ltd.; http://www.fluxus-engineering.com).

## Results

### Assay success rate

In total, 306 of the 384 (80%) SNPs scored successfully and were polymorphic (Data S1). Thirty-three SNPs were discarded because they either failed outright (22) or gave profiles that could not be scored (11). A further 45 SNPs were discarded because they amplified, but gave profiles that were monomorphic in the samples screened.

Of the 306 polymorphic SNPs for Eurasian beaver, 250 (79%) cross-amplified successfully in the Canadian beaver (scored in ≥3 beaver) and 8 (2.5%) were polymorphic. No markers were fixed for alternate alleles between the two species. A list of eight polymorphic SNP markers for *C. canadensis* can be found in Table S1.

Of the 321 animals screened, 279 had profiles which amplified for 95% or more of the 306 SNPs; these were taken forward for further analysis. A table of raw genotype data can be found in Table S2.

### Population genetic diversity

Polymorphism and heterozygosity at the 306 markers varied widely across populations (Table 3). Between 1 (Russia: Azas) and 92 (Germany: Bavaria)% of markers were polymorphic. Expected heterozygosity ranged from 0.04 (Russia: Konda and France) to 0.30 (Germany: Bavaria). Allelic richness estimated for a sample of ten individuals ranged from 1.1 (Russia: Azas) to 1.85 (Germany: Bavaria) alleles. The proportion of SNP markers showing departure from Hardy–Weinberg equilibrium (HWE) varied with population. Aside from Russia: Azas, of which 2 of the 4 of its polymorphic markers were out of HWE at the 5% level, Switzerland showed a high incidence of departure from Hardy–Weinberg equilibrium with 34% of its loci showing departures. The reason for this is apparent in the population structure analysis (below), which revealed animals from Switzerland to originate from multiple origins. For other populations, roughly 12% of the loci showed departure from Hardy–Weinberg (Table 3). The variability in the number of polymorphic markers discovered among populations undoubtedly reflects both genuine underlying differences in polymorphism and ascertainment bias (Morin et al. 2004; Chang et al. 2012) in the data set. Lower levels of polymorphism are found in some of the populations not represented in the RAD discovery phase (e.g. Russia: Konda/Azas, Mongolia: Bulgan). Pairwise Fst between all populations can be found in Table S4.

**Table 3:** Population-wide statistics for 306 SNP markers. Mean observed (H_O_) and expected (H_E_) heterozygosity with standard error, proportion of markers polymorphic, proportion polymorphic markers out of Hardy–Weinberg equilibrium (HWE) and allelic richness (rarefied to sample of 10 individuals) for sample locations for which *n* ≥ 10

	*n*	H_O_ (SE)	H_E_ (SE)	Proportion polymorphic	Proportion polymorphic out of HWE*	Allelic richness
Belarus	24	0.18 (0.010)	0.20 (0.010)	0.69	0.12	1.63
France	12	0.04 (0.008)	0.07 (0.007)	0.10	0.10	1.10
Germany: BWB	15	0.29 (0.012)	0.31 (0.010)	0.88	0.13	1.85
Germany: Bavaria	48	0.29 (0.011)	0.30 (0.010)	0.92	0.13	1.80
Germany: Hesse	16	0.16 (0.008)	0.17 (0.008)	0.77	0.11	1.70
Lithuania/Poland	40	0.24 (0.011)	0.25 (0.011)	0.77	0.12	1.71
Norway	48	0.14 (0.012)	0.14 (0.011)	0.38	0.14	1.36
Russia: Azas	12	0.01 (0.003)	0.04 (0.004)	0.01	0.50	1.01
Russia: Kirov	10	0.24 (0.013)	0.22 (0.011)	0.64	0.02	1.64
Russia: Konda	10	0.04 (0.008)	0.04 (0.007)	0.12	0.03	1.12
Russia: Voronezh	16	0.21 (0.014)	0.19 (0.011)	0.54	0.07	1.53
Switzerland	25	0.21 (0.008)	0.29 (0.010)	0.85	0.34	1.81

* *P* < 0.05, no Bonferroni correction applied.

### Visualizing the effects of ascertainment bias

Using principle component analysis (Genalex 6), clustering of the genotype data was analysed using four different overlapping SNP (sub)sets: all 306 SNPs, the ‘random’ panel (104 SNPs) and the panels that were chosen to be variable in Bavaria (84 SNPs) and Norway (81 SNPs) (Figure S2). The effects that ascertainment bias can have on SNP data are visually illustrated in Figure S2. In particular, the panels chosen specifically because they were polymorphic in either the Bavarian or Norwegian populations alter the scatter of the data with respect to the whole data set panels (all 306 and random 104 SNPs), with the difference being most noticeable in the Norwegian data set, where the variability in Norway is dramatically inflated versus the other populations. Additionally, the genetic distance of Norwegian populations to the Bavarian population is likely to be inflated as well. Therefore, we expect there to be a similar bias with respect to the populations that were not included in the RAD discovery phase (see later discussion).

### Population genetic structure at 104 SNP markers:

**Figure 3:**
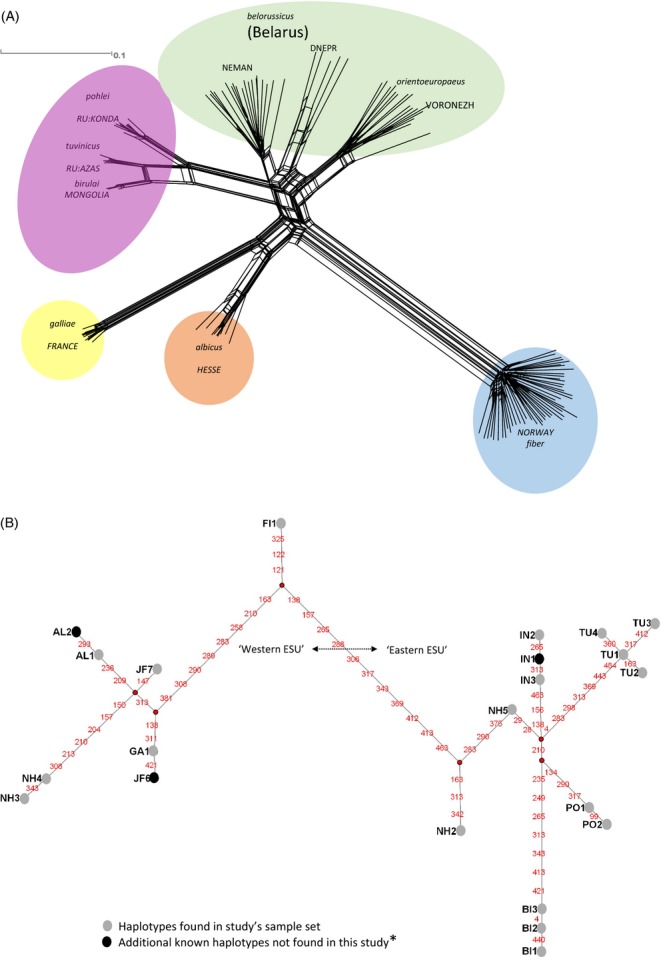
Phylogenetic structure of Eurasian beaver populations at 306 SNP. (A) A phylogenetic network of the SNP data assembled using the method neighbour-net (Bryant and Moulton 2004) in SPLITSTREE4 (Huson and Bryant 2006). Only populations thought to be ‘pure’ FT refugial populations are represented. For the population Germany: Hesse, individuals shown by the structure analysis to belong to other clusters have been removed. Clusters have been coloured according to the five major clusters that have been discovered by the STRUCTURE analysis. Animals from Belarus appear to divide into two separate clusters corresponding to different populations. Alongside these nuclear data (B) are a network of the mtDNA control region haplotypes known for *C. fiber* so far (Durka et al. 2005; Horn et al. 2010, this study). Haplotypes with prefixes AL, GA, FI, IN, PO and BI are from Durka et al. (2005). Haplotype JF7 is from Horn et al. 2010. Haplotypes prefixed ‘NH’ are new haplotypes discovered by this study. Division between ‘eastern’ and ‘western’ branches of the mtDNA phylogeny is well supported in both NJ and Bayesian analyses (see text). However, addition of the new samples appears to have broken down the east/west division because the haplotype JF7 which clusters on the ‘western’ branch originates from populations formerly classed as ‘eastern’. *See also recently published study by Horn et al. 2014 for additional, ancient, haplotypes.

In STRUCTURE, posterior likelihood (LnPD) showed a pattern of increase towards an asymptote with increasing *K* (Figure S1). Between *K* = 5 and *K* = 6, there was no significant increase in LnPD (*W* = 160, *P*-value = 0.2888), so *K* = 5 was chosen as the lowest value of *K* that captured the majority of the data structure. The five clusters which fell out consistently across all ten replicates in the following order were as follows:

1. Norway, 2. Central Eurasian populations (Russia: Azas, Russia: Konda and Mongolia: Bulgan) 3. France, 4. Germany: Hesse and 5. Eastern European populations (Fig. 2, Figure S3). These five clusters correspond approximately to the structure of PCA plots (Figure S2) and form monophyletic groups within the neighbour-net analysis (Fig. 3).

These population structure analyses of nuclear data reveal a number of factors about the Eurasian beaver which are briefly summarized here:

1. The Norwegian (*C. f. fiber*) population represents a distinct population that appears not to have introgressed with other European populations.
2. The French (*C. f. galliae*) population also represents a distinct population that appears not to have introgressed with other European populations.
3. Despite widespread reintroductions in Germany (Table 2), there does appear to be a distinct population of beaver in Hesse, reputedly the region where a remnant *albicus* subpopulation is located following transfer from its original location in the Elbe basin. Unfortunately, beavers still living in the actual Elbe basin were not tested as part of this study. Although animals from the Hesse region belong to a distinct cluster, introgression is also widespread with 43.8% of animals showing ≥5% introgression from other populations and a further two individuals (12.5%) clustering entirely with other populations (France, Eastern Europe and Norwegian clusters).
4. Beavers from Belarus (*C. f. belorussicus*) and Voronezh (*C. f. orientoeuropaeus*) in Russia appear to belong to a common genetic cluster and group with the populations from Lithuania and Poland and Russia Kirov, both reputedly mixed *belorussicus* and *orientoeuropaeus* (Table 2). In the STRUCTURE analysis with higher values of *K* and in the network and PCA analysis, further subdivision between these regions is observable, with Voronezh (*orientoeuropaeus*) falling out at *K* = 6 and a Lithuania/Poland cluster at *K* = 7. At *K* = 8, a clear split within the Belarus (*belorussicus)* populations becomes apparent (Figures S2 and S3). This split can also be seen in Fig. 3.
5. Russia: Azas, Russia: Konda and Mongolia: Bulgan group as close but distinct populations in neighbour-net and PCA analyses (Fig. 3, Figure S2), whereas in the STRUCTURE analysis, they cluster as a single group (this holds true up to *K* = 8, Figure S3). Due to the experimental design of the RAD sequencing project (see above), ascertainment bias is likely to be present and may strongly affect the results for these Central Eurasian populations (discussed later).
6. Beavers from Bavaria in Germany are clearly of admixed descent, with apparent nuclear DNA ancestral contributions from France and Eastern Europe clusters, although interestingly no Norwegian contribution (Table 2). All individuals have membership to both population clusters indicating that admixture occurred a number of generations previously and genes from both parent populations have subsequently spread through the introduced population (SNP markers in this population generally also conform to HWE, Table 3). The population in Baden-Württemberg is also of similar origin although two animals clearly show nuclear introgression from Norway (>5%) that has not been found in Bavaria (both individuals come from Waldshut in Baden-Württemberg). This is presumably due to the close proximity of the Swiss population (see below).
7. Beavers in Switzerland show multiple genetic origins in accordance with their purported population history (Table 2), with animals either belonging predominantly to the French cluster (*n* = 6, ≤5% introgression), or showing introgression between Norwegian, Eastern European and French clusters. The six animals that belonged entirely to the French cluster came from the Rhône watershed area in the Swiss cantons of Geneva, Vallais and Vaud and are presumably immigrants from France. Hardy–Weinberg disequilibrium in the Swiss ‘population’ is high for the reason that it actually spans a number of disconnected populations. Fine-level population structure was not the purpose of this study, but see Frosch et al. (2014).

### MtDNA analysis and cytonuclear concordance:

**Figure 4:**
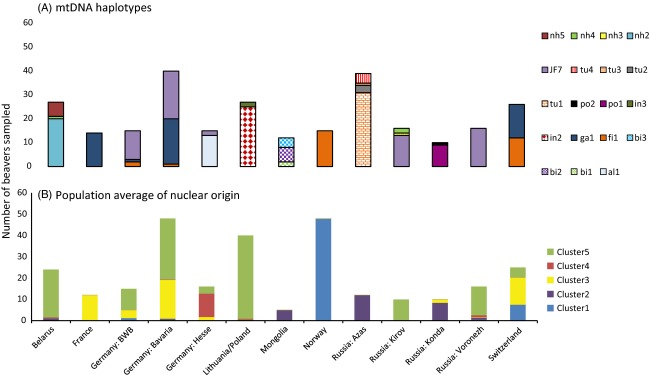
Count of control region haplotypes (A) in 245 beavers (a subset of the samples placed on the SNP chip) screened at 490 bp of mtDNA control region, in comparison with (B) the average assignment to STRUCTURE cluster at 104 SNP loci for the main populations investigated. Haplotype diversity per population is low, with the populations of France and Norway exhibiting only a single haplotype. European population of that are shown to be mixed origin at SNP markers also show multiple haplotypes (German populations and Switzerland). In general, nuclear and mitochondrial data agree. Noticeable is the fact that the Central Eurasian populations contain higher haplotype diversity than suggested by the SNP data.

In addition to the haplotype survey previously published by Durka et al. (2005), an additional five haplotypes (jf7, nh2-5) were discovered spread across the following 6 populations: Germany: (Bavaria, Baden-Württemberg henceforth ‘BWB’, Hesse), Belarus, Russia (Kirov, Voronezh) (Figs 3 and 4). Jf7 has been previously published (Horn et al. 2010) as Jf264887.1 in a beaver of uncertain origin sampled near Berlin, Germany. These discoveries were all in populations not previously surveyed by Durka et al. (2005).

Topology under the two tree building methods (neighbour-joining, MrBayes) was broadly concordant. Under both analyses ‘eastern’ (po1-2, in1-3, bi1-3 and tu1-4) and ‘western’ (al1, al2, ga1, fi1) haplotypes original to the Durka et al. (2005) papers clustered on separate branches with 100% bootstrap support/posterior probability of 100%. However, not all the haplotypes found additionally (jf7, nh2-nh5) conformed to the previously reported east/west division (Durka et al. 2005). Haplotype nh2 and haplotype nh5 that were found in Belarus and grouped, as expected, within the eastern clade; however, haplotype jf7, found at an incidence of 100% in the putative *orientoeuropaeus* FT refugia of Voronezh, the population in Kirov (mixed *orientoeuropaeus* and *belorussicus*, 81.3%) and the reintroduced populations of Germany: Bavaria (50%), BWB (80%) and Hesse (10%), grouped within the western clade (Fig. 4). In addition, haplotype nh4, found in Belarus at an incidence of 4% (one individual), also grouped with the western clade.

A network of the haplotypes reveals that there were 42 mutational steps needed to move across extremes of the network from haplotype nh3 to bi1 (Fig. 3). However, the actual number of mutations between these two haplotypes was considerably less at 23 (equating to 4.7% divergence) suggesting that there are a number missing links within the network and that it is poorly resolved.

### Power for parentage analysis and individual ID

The examination of the power of the markers to perform parentage and individual identification was conducted for all the main populations (Belarus, France, Germany: BWB; Germany: Bavaria, Lithuania and Poland; Mongolia; Norway; Russia: Azas, Konda, Kirov and Voronezh). In all of the populations except Mongolia and Russia: Azas, a panel of 20 or fewer markers was sufficient to have a probability of identity (PID) of <1/10 000. This increases to a panel of 30 or fewer assuming a population of full sibs (PID_SIB) (Sup.Mat 4). Due to the low number of polymorphic markers in Mongolia and Russia: Azas, the maximum achievable PID in these populations was 0.00712 (7 markers) and 0.09879 (four markers), respectively. In all but Mongolia, Russia: Azas, Russia: Konda and France, 30 or fewer markers was sufficient for parentage assignment (probability of excluding both false parents >0.9998). Tables of probabilities and markers suitable for addressing parentage and individual ID questions in each population are available in Table S3.

## Discussion

### The purpose of conducting genetic analysis in support of reintroductions

There are two predominant reasons why it is desirable to conduct genetic analysis in support of population reintroduction and augmentation work:

1. To aid with the selection of founder population(s) and individuals from within these population(s) to be released.
2. To conduct ongoing management and postrelease monitoring of population.

The second consideration, the postrelease monitoring of individual animals, requires markers that are sufficiently polymorphic to conduct individual identification and parentage analysis. This study discovered that small panels of SNP markers have sufficient power to perform this function in most target populations of interest for the Eurasian beaver. In most populations, panels of 20–30 markers should be sufficient to address parentage and individual ID questions at a level of exclusion probability suitable for ecological monitoring (<1/10 000) (Table S3). Variation in the number of markers required for different populations will reflect both genuine underlying differences in genetic diversity and ascertainment bias of the selected panel of 306 (see later). Markers used for monitoring should ideally be suitable for use on noninvasive samples (e.g. hair traps, faecal samples). The Illumina Vercaode SNP assay used here is not well suited to poor quality sample types as it requires high concentrations of DNA (>25–50 ng^−1^ *μ*l), but uniplex SNP assays or approaches using rapid parallel SNP genotyping based on nanofluidic dynamic arrays (Wang et al. 2009) are. SNPs also have an advantage over the more traditionally used microsatellites in that the data sets require no calibration between laboratories (Vignal et al. 2002; Morin and Mccarthy 2007). This is a crucial consideration when working on conservation projects where the responsibility for genetic monitoring may pass between different laboratories over time. A disadvantage that the use of SNPs may have over microsatellites is a decreased ability to resolve fine-scale population structuring (DeFaveri et al. 2013).

The first consideration, to aid with the selection of source populations and individuals from within these populations for reintroduction, is more complex. There are three primary genetic considerations when choosing animals for a reintroduction, which may not always be synergistic:

1. To select individuals with low levels of inbreeding and high combined genetic diversity.
2. Conversely, to ascertain that the introduced combination of animals is not likely to suffer outbreeding depression.
3. To select the most similar individuals to those historically present (IUCN 1998; IUCN/SSC 2013).

These issues will now be examined in turn with respect to the results of this study.

### To select individuals with low inbreeding coefficients and high genetic diversity

Arguably, the most important consideration is choosing individuals with low inbreeding coefficient and a high combined genetic diversity. Most animals of conservation concern either currently exist in small populations or have come from populations that have previously undergone a bottleneck (as in this example of the Eurasian beaver) and so are subject to high levels of inbreeding. Although the possibility of inbreeding is often skimmed over by conservation practitioners [as illustrated by the fact that 37% of published reintroduction studies report fewer than twenty founding animals or fail to report the number of founders (Fischer and Lindenmayer 2000)], inbreeding has been shown on countless occasions to have a detrimental effect on fitness in naturally outbreeding species (Darwin 1876; Crnokrak and Roff 1999; Spottiswoode and Møller 2004) and review by Frankham (2010). This includes studies of captive populations (Ralls and Ballou 1983) and in populations released into the wild (Frankham 1995; Kephart 2004; Vilas et al. 2006). Therefore, to increase the success of a reintroduction, efforts should be made to minimize the level of inbreeding in the reintroduced individuals and also to minimize inbreeding postrelease (remedial actions postrelease could involve the augmentation of founders, translocation of individuals between physical localities or the facilitation of metapopluation joining).

Selection of founder individuals from a single population to minimize inbreeding should ideally be conducted using (genetically verified) pedigree data, not measures of genetic diversity (e.g. heterozygosity) alone, especially if only few genetic markers are available (Balloux et al. 2004; Pemberton 2004; Slate et al. 2004). However, if pedigree data are missing, incomplete or base levels of inbreeding are high, and then, heterozygosity (measured at a large number of loci) is a more appropriate measure (Bensch et al. 2006; Ruiz-López et al. 2012; Townsend and Jamieson 2013). As the option of sourcing Eurasian beaver from the wild is available (and indeed preferable; IUCN 1998, 2013), we have used a suite of 306 SNP markers to first survey the genetic diversity and relatedness of candidate source populations to provide the background information to aid with founder population selection. Once founder population have been decided upon, founder candidate individuals should be screened for heterozygosity and pairwise relatedness using molecular measures, as pedigree data will not be available. In addition, this will generate the baseline data for future individual-based monitoring.

Related to the issue of inbreeding is the maximization of genetic diversity within the founder stock. This is typically measured, as in this study, by allelic richness and/or heterozygosity at neutral or randomly selected loci. These measures are taken as a proxy for the degree of underlying adaptive variation conferred by an assortment of unknown genes located throughout the genome (Ouborg et al. 2010). This maximization of genetic diversity is desirable in order to maximize the adaptive potential of the population, so that it has the capacity to evolve – to be resilient to future environmental change, disease, and to retain the ability of being able to readapt to the wild environment from captivity (Christie et al. 2012).

In the case of the Eurasian beaver, one pattern appears to be clear. The genetic diversity in the mixed, reintroduced populations (Bavaria, Switzerland, Baden-Württemberg) is higher than in the FT refugial populations (France, Norway, Hesse) (Table 3). This finding is confirmed by Frosch et al. (2014), who used microsatellite analysis to compare genetic diversity in pure versus admixed beaver populations. However, the discovery of the SNP markers used in this study, based on a small number of animals from Norway and Bavaria (Senn et al. 2013) has undoubtedly introduced ascertainment bias to the results (Albrechtsen et al. 2010). This will almost certainly make the estimates of genetic diversity in the far eastern (central Eurasian) populations unreliable as they are distantly related to the SNP discovery populations. Bavarian animals have been shown to have nuclear genetic contribution from both Eastern European and French clusters (Fig. 2), and the inclusion of these animals within the RAD sequencing project (Senn et al. 2013) should ameliorate the effect of ascertainment bias on estimates of diversity in these populations, although there is still a question mark over to what extent it may be intensifying the results of low genetic diversity in the *galliae* (French) and *albicus* (Germany: Hesse) samples (Table 3) (further hampered by the small sample sizes for these populations). Addition of further RAD sequencing data from these underrepresented populations to that produced by Senn et al. (2013) would allow for the selection of a new panel of markers more suitable for use across all populations in future. Alternatively a direct genotyping-by-sequencing (Narum et al. 2013) approach could be chosen in future to avoid worries about ascertainment bias. Despite these caveats, the patterns in genetic diversity that we uncover here suggest that the possibility of mixing founding stock is an important consideration for this reintroduction study. This leads on to a second consideration:

### To ascertain that the introduced combination of animals is not likely to suffer outbreeding depression

Frankham et al. (2011) argues that when considering the option of mixing different populations for conservation purposes, the risk of outbreeding is generally much lower than the risk of inbreeding, but that it is the former risk that conservation practitioners tend to worry most about. Predicting the probability of outbreeding depression in advance is not an easy task and they suggest a flow chart for evaluating risk, where the risk of outbreeding depression is smaller with the absence of chromosomal differences, absence of gene flow for >500 years and lack of substantial environmental differences between the populations (Fig. 1 of Frankham et al. 2011).

In the case of the Eurasian beaver, karyotype differences are thought to be absent (Graphodatsky et al. 1991, Lavrov and Orlov 1973). ‘Major environmental difference’ is not easy to quantify, but Eurasian beaver are thought to differ little in physiology, behaviour or habitat preference and the environments that they inhabit are broadly similar (Heidecke 1986; Rosell et al. 2005). They are, however, reliably distinguishable only by detailed morphometric comparisons and via, karyotypic, biochemical or molecular measurements from the North American *C. canadensis* (Rosell and Sun 1999; Kuehn et al. 2000; Dewas et al. 2012; McEwing et al. 2014) with which they are not interfertile. So, given that it is not easily distinguishable from its sister species, broad phenotypic similarity within Eurasian beaver cannot necessarily be taken as a sign that reproductive isolation/incompatibility is completely absent given that it is not easily distinguishable from its next closest relative.

The issue of the extent to which Eurasian beaver populations are related was first examined by Durka et al. (2005) using the mtDNA control region data. They discovered monophyletic clades at the mtDNA control region dividing *C. fiber* into an ‘eastern’ and ‘western clade’ (Table 1), which qualified as evolutionary significant units (ESU) according to the criteria of Moritz (1994) (but see also Paetkau 1999; Fraser and Bernatchez 2001 for criticism of ESU). The most recent common ancestor in Eurasian beaver has been dated to around 210 000 years ago (Horn et al. 2011) and it is likely that much subsequent divergence was driven by glaciation (Durka et al. 2005). Durka et al. (2005) also argued, however, that reciprocal monophyly of the eastern and western populations could also have developed in the last few hundred years as a result of drastic population fragmentation and bottlenecking due to the fur trade, but suggested that two clades should be treated as separate management units until further genetic evidence to the contrary arose. A recent study of ancient mtDNA by Horn et al. (2014) has indeed demonstrated that strong apparent phylogeographic structuring in Eurasian beaver has arisen as a result of the population bottlenecks, although they suggest that eastern and western ESUs are maintained. This analysis both examines the original samples of Durka et al. (2005) at the nuclear SNP markers and adds to the data set, critically with two eastern FT refugial populations not previously sampled: *C. f. belorussicus* (Belarus) and *C. f. orientoeuropaeus* (Voronezh). While the phylogenetic tree of the nuclear data (Fig. 3) broadly corresponds to the original mtDNA picture, it of course inevitable that it is subject to the same bottle-necking effects from near extirpation by hunting as the mtDNA data (Horn et al. 2014). The additional mtDNA data, does however, suggest that a deep east/west spilt is not as apparent as previously thought as the *C. f. orientoeuropaeus* FT refugial population (Voronezh), which should group geographically within the eastern populations according to Durka et al. (2005) (see Figs 1) and groups unambiguously with Eastern European beavers at nuclear loci, actually has a mtDNA haplotype that is from the putative ‘western’ clade (Fig. 5). Additionally, the more westerly situated population within Belarus (*putative C. f. belorussicus*) exhibits a mixture of haplotypes that span the putative east/west division (Figs 3 and 5). Taken together, this evidence suggests that the division between eastern and western ESU is not as distinct as laid out by Durka et al. (2005) and that the conditions of reciprocal monophyly may have been broken. A likely suggestion is that divergence in mtDNA haplotypes did indeed arise following population retreat into glacial refugia during that last glacial maxima (∼25 000 ya), but that introgression following secondary contact of re-emergent populations caused subsequent mixing of divergent haplotypes in contact regions (located in Eastern Europe). Further studies with more extensive contemporary and historical coverage of possible ESU boundary areas would be required to investigate this issue more fully.

We suggest, however, that based on the available evidence, there is limited phylogenetic justification for postulated ESU or subspecies divisions (see also Horn et al. 2014 for opinions). The traditional FT refugial populations (Table 1) are undoubtedly valuable repositories of genetic diversity as indicated by their divergence both at nuclear and mtDNA markers, but the patterns of divergence are not consistent with total isolation. We also point the reader at this point to a thorough criticisms of the use of phylogenetic data to justify taxonomic inflation and its effect on conservation (Frankham et al. 2012; Zachos et al. 2013).

Additionally and perhaps more pragmatically, the data provided by this study can make some evaluations from the admixture experiments that have already been run. Divergence at neutral loci has been shown to be a poor indicator of outbreeding depression in experimental crosses of fish (McClelland and Naish 2006), and the same may well be the case for other species. Although we cannot assess the direct fitness consequences of crossing beaver here, the genetic legacy of past reintroduction can act as a limited kind of experimental evidence. We know that beavers from putative *fiber, belorussicus* and *galliae* subspecies were introduced to Bavaria (Table 2). The genetic evidence from this study confirms that mixing of French and Eastern European and to a lesser extent Norwegian gene pools has occurred in Bavaria leading to a formation of a stable admixed population (as indicated by the relatively even contributions to each cluster of the individuals in those populations, Fig. 2). The populations in Switzerland, purportedly originating from Norway (*fiber*), Russia (Voronezh) and French stock, show more recent signs of admixture between the expected population clusters (French, Norwegian and Eastern European) as indicated by the more variable contribution from each cluster within admixed individuals (Fig. 2) and deviation from HWE. Many of these individuals clearly show more complex ancestry than that of first generation crossing between groups indicating that, again, interpopulation fertility is present. A signature of admixture does not of course mean that a certain proportion of the population has not suffered from outbreeding depression. Despite only minor variations in phenotype between *C. fiber* populations, differentiation in chemical signalling has been found: Norwegian beavers from Telemark respond more strongly to castoreum from other Telemark beavers than to that from Elbe beavers in Germany (Rosell and Steifetten 2004), suggesting that some level of premating isolation between populations occurs. However, this is apparently not strong enough to have prevented all cross-breeding with Norwegian beavers (see for example Switzerland, Fig. 2).

If mild outbreeding depression occurs when populations are mixed, it is likely that, in time, natural selection will act on the elevated genetic diversity within the gene pool to eliminate it (Edmands et al. 2005; Erickson and Fenster 2006). The other options for outcome are extinction or formation of a stable ‘hybrid’ zone at the boundary of mixing (Barton and Hewitt 1985). The evidence in Bavaria where there are an estimated 14–6000 (Schwab 2013; Frosch et al. 2014) beavers indicates that neither of these latter two scenarios has occurred. To avoid possible issues with outbreeding depression within the beaver founding stock, if mixing were a strategy to be pursued, one option might be to source beavers for the reintroduction from genetically diverse premixed population that has already passed through a number of generations of natural selection (for example Bavaria or Switzerland).

The final question to be discussed is regarding the choice of animals similar to the historical population.

### To select the most similar individuals to those historically present

The original IUCN guideline for reintroduction (IUCN 1998) states that, ‘If there is a choice of wild populations to supply founder stock for translocation, the source population should ideally be closely related genetically to the original native stock and show similar ecological characteristics (morphology, physiology, behaviour, habitat preference) to the original subpopulation.’ The updated 2012 version (IUCN 2013) softens this statement: ‘Founders should show characteristics based on genetic provenance, and of morphology, physiology and behaviour that are assessed as appropriate through comparison with the original or any remaining wild populations…’.

The underlying reason for choosing animals most genetically similar to the original population for a reintroduction should be to maximize the chance of the variation present in the reintroduced population being adaptive to the reintroduction site. However, closely related populations (if measured genome wide and at neutral loci) may not share the same adaptive traits (for example, if they exist in very different environmental conditions), and distantly related populations may evolve similarly adaptive traits in parallel. In the case of the British population of the Eurasian beaver, which went extinct in the 16th century (Kitchener and Conroy 1997 and references therein), it has not yet been possible to make a direct genetic comparison. The issue regarding which extant population is most closely related to the original population in Britain has been examined using cranial evidence [a sample set of 108 crania and mandibles from British beavers measured at 21 measurements which showed greatest similarity to animals from Norway (Kitchener and Lynch 2000)]. Similarity in morphology may, however, be due to environmental factors and not relatedness. Arguments based on evidence of postglacial colonization routes also offer no clearer resolution. The most likely origin of beavers in Britain was through recolonization around 10 000 years ago once the climate had warmed sufficiently to create suitable habitat following the last glacial maxima (Coles 2006), but before sea levels rose to cut-off Britain from the European mainland (∼8000 BP). There were therefore theoretically three possible colonization routes open: 1. from Eastern Europe via the North Sea, 2. from France across the English Channel and 3. from Germany via the southern North Sea. Multiple routes may have been taken as in the case of the postglacial colonization of the water vole of Britain (Piertney et al. 2005; Coles 2006). The cranially similar Scandinavian populations may have in turn arrived in Southern Norway either via an Eastern European or French route, as crossing would have been possible over the land bridge between Denmark and Sweden which persisted until 7500 BP (Kitchener and Lynch 2000; Halley 2011). Although this question of colonization route and therefore the origin of the British beaver may be resolvable using DNA from museum specimens, it may not be the most pertinent issue when it comes to ongoing conservation efforts in Britain. Given the ever present possibility of climate change, the potential for a population to adapt widely as opposed to it possessing the best adaptive fit to (past) environment is arguable a more important consideration (Broadhurst et al. 2008; Sgrò et al. 2011).

## Conclusions

Halley (2011) suggested three possible founder options for a British reintroduction: unmixed stock from a single western FT refugia, mixed western ESU stock or a mixed eastern and western ESU FT refugial stock. This study has demonstrated through additional sampling and nuclear genetic analysis that the ESU division suggested by Durka et al. (2005) is not as obvious as previously thought. Through the use of nuclear genetic data we have confirmed that reintroductions stemming from mixed population founder stock, do in fact have mixed genetic heritage, further supporting the possibility that interbreeding between FT refugial populations can occur and does not result in a major loss of fitness. We have additionally demonstrated that genetic diversity is considerably lower in FT refugial populations than those from mixed founders. For the arguments given in the above sections, we suggest the risks of inbreeding depression during a reintroduction are likely to be much higher than outbreeding depression (Frankham et al. 2011; Weeks et al. 2011). Indeed the risks of inbreeding are high even when choosing genetically diverse founding stock, as reintroductions will pass through a bottleneck due to founder effects associated with population subsampling and postrelease mortality. These effects can be ameliorated by the release of large numbers of founding individuals (Fischer and Lindenmayer 2000).

In conclusion, we suggest that the first scenario (the use of unmixed stock) is the least desirable in view of the low genetic diversity in FT refugia populations, although we underline that there is no experimental evidence available for inbreeding depression in beaver and highly bottlenecked, single source populations exist apparently successfully. We also conclude that there is no genetic evidence to preclude either of the two remaining scenarios proposed by Halley (2011) as we see evidence of both historical and current mixing of the postulated ESU. The ‘ideal world’ scenario is to take animals from a genetically diverse source that is also closely related to the original population. The final choice must balance the need for genetic diversity against phylogenetic fit, a dilemma that is faced in all reintroductions.

Genetic considerations are not the only consideration when choosing founders for a reintroduction. Decisions must be made based on availability of source animals, the impact of removing of the founding animals from the source population, animal welfare, veterinary and ecological consideration (IUCN 1998, 2013). Reintroduction should be publically supported, conducted legally, risk assessed, planned and implemented using best available scientific data and carried out with a commitment to ongoing monitoring (IUCN 1998, 2013; Fischer and Lindenmayer 2000). All else being equal, using a genetically diverse founder stock of a large number of animals, that is, monitored for inbreeding following release, represents the lowest risk genetic strategy for ensuring the long-term survival of the reintroduction.

## Data accessibility

DNA sequences: GenBank accessions KJ670496–9. Remaining data: as supplementary material.

## Supporting Information

**Figure S1.** Value of LnPD against *K* for ten replicates of STRUCTURE output.

**Figure S2.** Principle Component Analysis of beaver SNP data sets.

**Figure S3.** Graphical representation of structure output *K* = 2–8, populations numbered as in Table 2.

**Data S1.** List of SNPs

**Table S1.** List of Canadian beaver SNPs.

**Table S2.** Raw genotype data.

**Table S3.** Panels of markers required for parentage identification and individual id.

**Table S4.** Pairwise FST.
