# Supplementary material for: Nuclear and mitochondrial genetic structure in the Eurasian beaver (Castor fiber) – implications for future reintroductions
Source: Evol Appl. 2014 Jun 17;7(6):645–62. doi: 10.1111/eva.12162 (PMC4105916; doi:10.1111/eva.12162)
Supplement: Supplementary file 1 — Figure S1. Value of LnPD against K for ten replicates of STRUCTURE output. [file eva0007-0645-SD1.pdf]

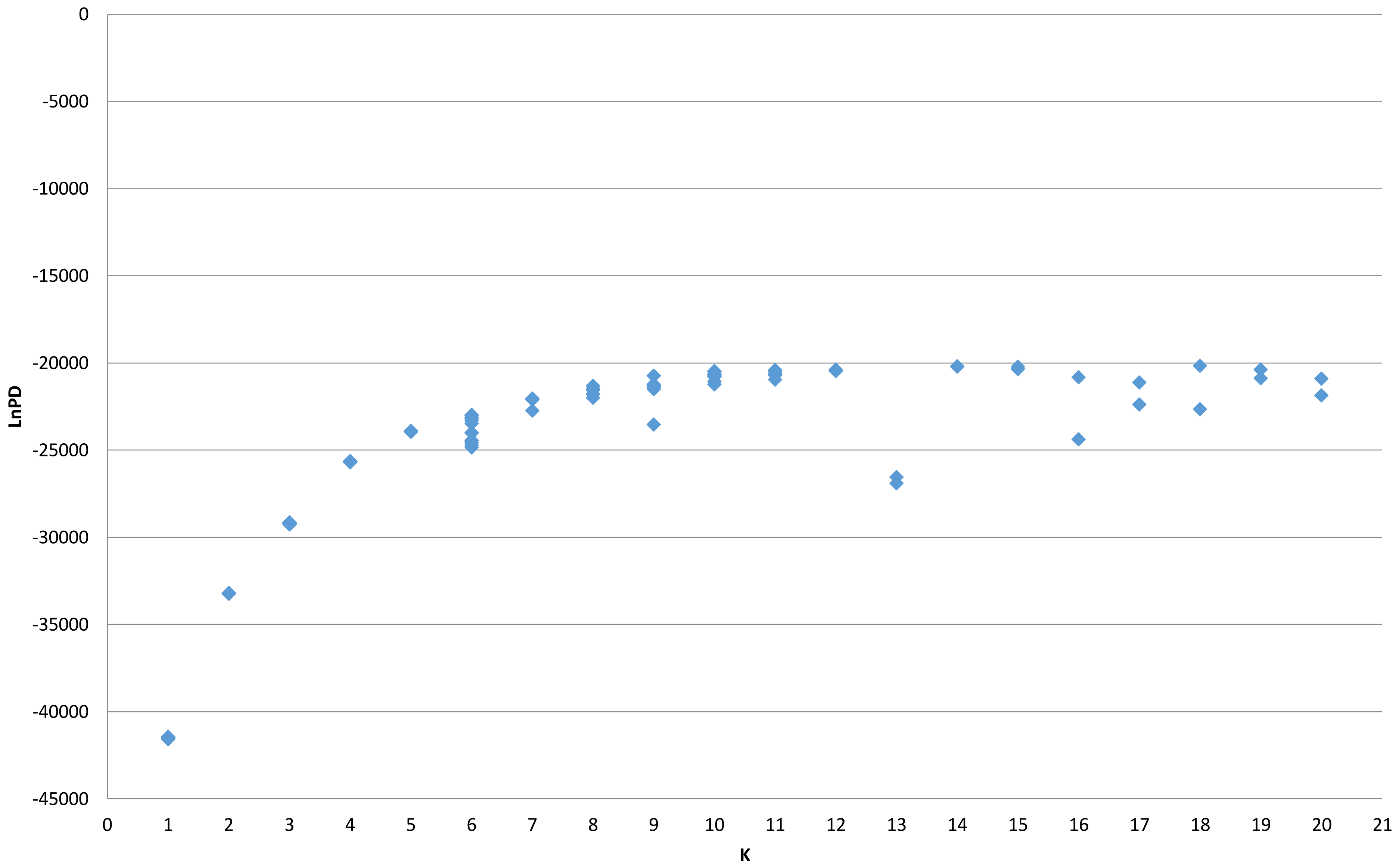

Supplementary Material Figure1: Value of LnPD against K for ten replicates of Structure output.
