# Supplementary material for: Nuclear and mitochondrial genetic structure in the Eurasian beaver (Castor fiber) – implications for future reintroductions
Source: Evol Appl. 2014 Jun 17;7(6):645–62. doi: 10.1111/eva.12162 (PMC4105916; doi:10.1111/eva.12162)
Supplement: Supplementary file 2 — Figure S2. Principle Component Analysis of beaver SNP data sets. [file eva0007-0645-SD2.pdf]

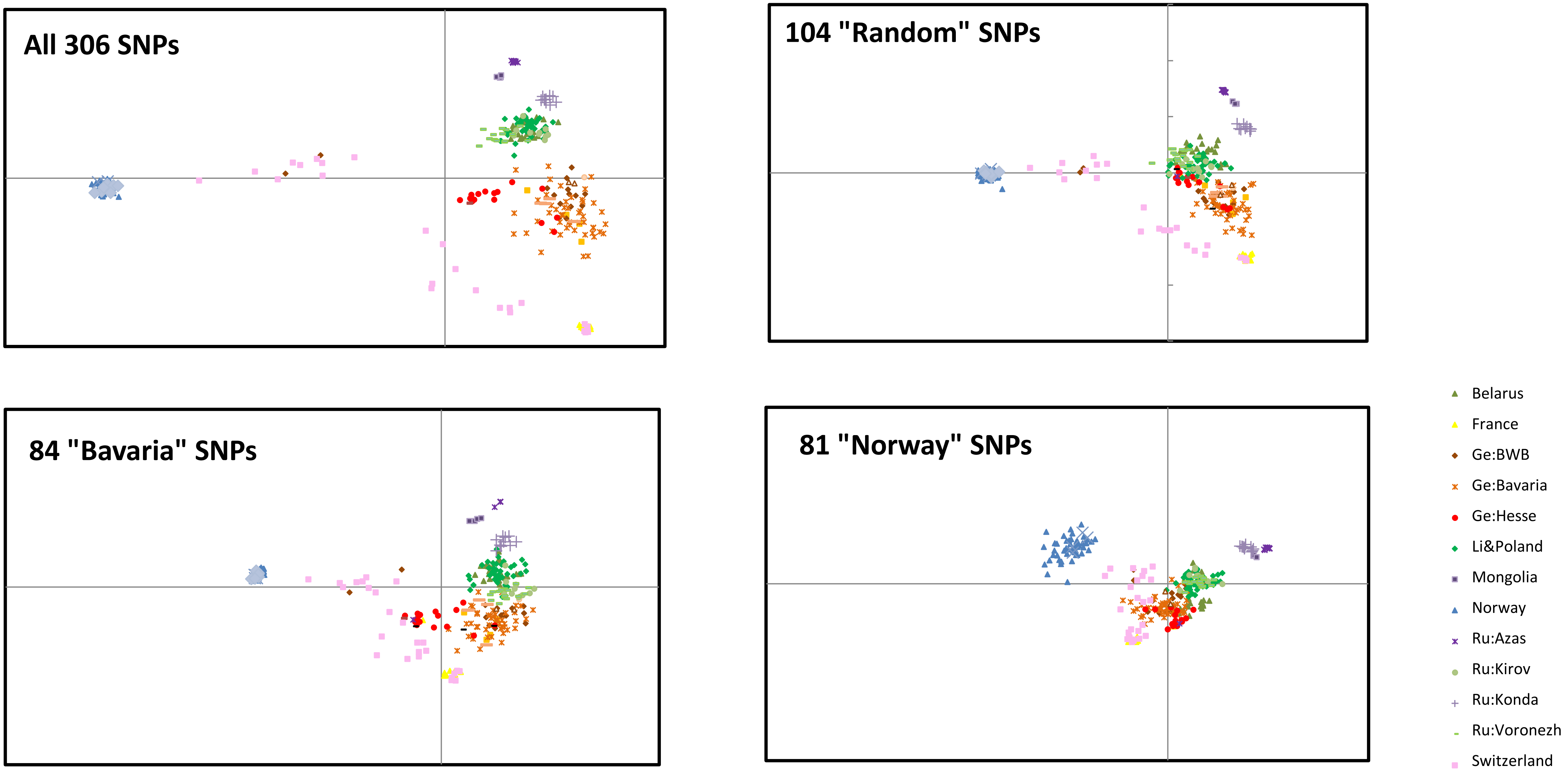
All 306 SNPs
104 "Random" SNPs
84 "Bavaria" SNPs
81 "Norway" SNPs
Belarus
France
Ge:BWB
Ge:Bavaria
Ge:Hesse
Li&Poland
Mongolia
Norway
Ru:Azas
Ru:Kirov
Ru:Konda
Ru:Voronezh
Switzerland
