# Supplementary figures and images for: Nuclear and mitochondrial genetic structure in the Eurasian beaver (Castor fiber) – implications for future reintroductions
Source: Evol Appl. 2014 Jun 17;7(6):645–62. doi: 10.1111/eva.12162 (PMC4105916; doi:10.1111/eva.12162)

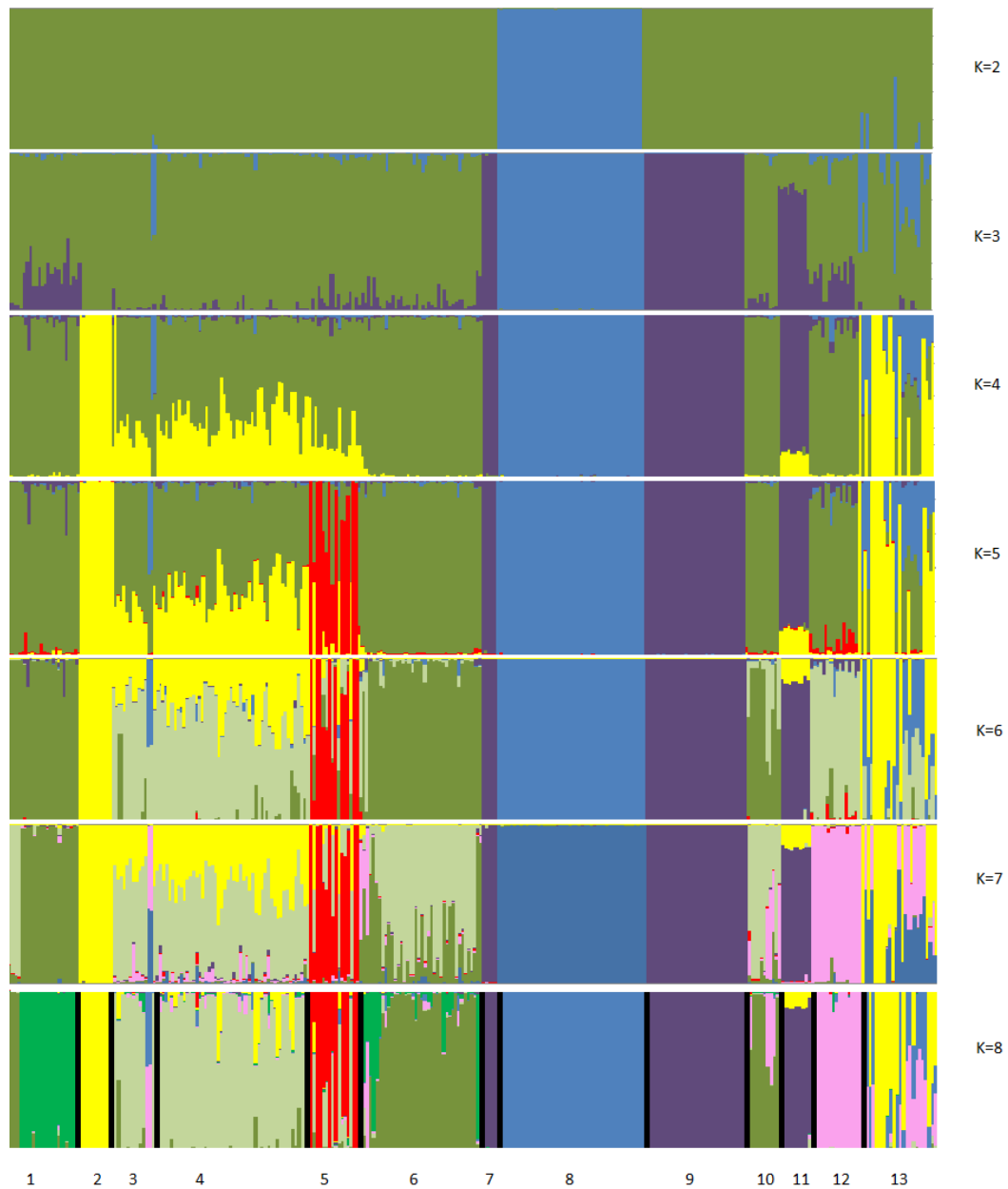

Supplement: Supplementary file 3 — Figure S3. Graphical representation of structure output K = 2–8, populations numbered as in Table 2. [file eva0007-0645-SD3.pdf]
