# Supplementary material for: Nuclear and mitochondrial genetic structure in the Eurasian beaver (Castor fiber) – implications for future reintroductions
Source: Evol Appl. 2014 Jun 17;7(6):645–62. doi: 10.1111/eva.12162 (PMC4105916; doi:10.1111/eva.12162)
Supplement: Supplementary file 8 — Table S4. Pairwise FST. [file eva0007-0645-SD8.docx]

| Belarus | France | Germany: Baden-Württemberg | Germany: Bavaria | Germany: Hesse | Lithuania and Poland | Mongolia | Norway | Russia: Azas | Russia: Kirov | Russia: Konda | Russia: Voronezh | Switzerland |  |
| --- | --- | --- | --- | --- | --- | --- | --- | --- | --- | --- | --- | --- | --- |
| 0.000 |  |  |  |  |  |  |  |  |  |  |  |  | Belarus |
| 0.328 | 0.000 |  |  |  |  |  |  |  |  |  |  |  | France |
| 0.103 | 0.209 | 0.000 |  |  |  |  |  |  |  |  |  |  | Germany: Baden-Württemberg |
| 0.110 | 0.161 | 0.022 | 0.000 |  |  |  |  |  |  |  |  |  | Germany: Bavaria |
| 0.149 | 0.251 | 0.118 | 0.113 | 0.000 |  |  |  |  |  |  |  |  | Germany: Hesse |
| 0.047 | 0.317 | 0.066 | 0.079 | 0.144 | 0.000 |  |  |  |  |  |  |  | Lithuania and Poland |
| 0.270 | 0.805 | 0.263 | 0.268 | 0.293 | 0.251 | 0.000 |  |  |  |  |  |  | Mongolia |
| 0.318 | 0.562 | 0.278 | 0.310 | 0.298 | 0.300 | 0.560 | 0.000 |  |  |  |  |  | Norway |
| 0.260 | 0.831 | 0.265 | 0.269 | 0.317 | 0.244 | 0.712 | 0.578 | 0.000 |  |  |  |  | Russia: Azas |
| 0.102 | 0.363 | 0.080 | 0.091 | 0.160 | 0.054 | 0.325 | 0.332 | 0.314 | 0.000 |  |  |  | Russia: Kirov |
| 0.245 | 0.665 | 0.223 | 0.225 | 0.279 | 0.224 | 0.519 | 0.536 | 0.562 | 0.286 | 0.000 |  |  | Russia: Konda |
| 0.168 | 0.422 | 0.115 | 0.130 | 0.182 | 0.129 | 0.376 | 0.350 | 0.363 | 0.090 | 0.346 | 0.000 |  | Russia: Voronezh |
| 0.155 | 0.141 | 0.082 | 0.082 | 0.127 | 0.144 | 0.313 | 0.222 | 0.325 | 0.161 | 0.275 | 0.176 | 0.000 | Switzerland |
